# Characterization of clinical *Ralstonia* strains and their taxonomic position

**DOI:** 10.1007/s10482-021-01637-0

**Published:** 2021-08-31

**Authors:** Ad C. Fluit, Jumamurat R. Bayjanov, María Díez Aguilar, Rafael Cantón, Michael M. Tunney, J. Stuart Elborn, Mireille van Westreenen, Miquel B. Ekkelenkamp

**Affiliations:** 1 Department of Medical Microbiology, University Medical Center Utrecht, Room G04.614, PO Box 85500, 3508 GA Utrecht, The Netherlands; 2 Servicio de Microbiología, Hospital Universitario Ramón y Cajal and Instituto Ramón y Cajal de Investigación Sanitaria (IRYCIS), Madrid, Spain; 3 Red Española de Investigación en Patología Infecciosa (REIPI), Madrid, Spain; 4 Halo Research Group, Queen’s University Belfast, Belfast, UK; 5 Department of Medical Microbiology, Erasmus University Medical Center, Rotterdam, The Netherlands

**Keywords:** Cystic fibrosis, Infection, *Ralstonia*, Taxonomy, Whole genome sequence

## Abstract

To improve understanding of the role of *Ralstonia* in cystic fibrosis (CF), whole genomes of 18 strains from clinical samples were sequenced using Illumina technology. Sequences were analysed by core genome Multi-Locus Sequence Typing, Average Nucleotide Identity based on BLAST (ANIb), RAST annotation, and by ResFinder. Phylogenetic analysis was performed for the 16S rRNA gene, and the OXA-22 and OXA-60 ß-lactamase families. The minimal inhibitory concentrations (MICs) were determined using broth microdilution. ANIb data for the 18 isolates and 54 strains from GenBank, supported by phylogenetic analysis, showed that 8 groups of clusters (A-H), as well as subgroups that should be considered as species or subspecies. Groups A-C contain strains previously identified as *Ralstonia solanacearum* and *Ralstonia pseudosolanacearum*. We propose that group A is a novel species. Group B and C are *Ralstonia syzygii*, *Ralstonia solanacearum*, respectively. Group D is composed of *Ralstonia mannitolilytica* and Group E of *Ralstonia pickettii*. Group F and G should be considered novel species. Group H strains belong to *R. insidiosa*. OXA-22 and OXA-60 family ß-lactamases were encoded by all strains. Co-trimoxazole generally showed high activity with low MICs (≤1 mg/l) as did ciprofloxacin (≤0.12 mg/l). MICs against the other antibiotics were more variable, but generally high. RAST annotation revealed limited differences between the strains, and virulence factors were not identified. The taxonomy of the genus *Ralstonia* is in need of revision, but sequencing additional isolates is needed. Antibiotic resistance levels are high. Annotation did not identify potential virulence factors.

**Supplementary Information:**

The online version contains supplementary material available at 10.1007/s10482-021-01637-0.

*Ralstonia*, a genus of Gram-negative bacteria belonging to the family Burkholderiaceae first described in 1995 (Yabuuchi et al. 1995), is currently comprised of six species: *Ralstonia insidiosa*, *Ralstonia mannitolilytica*, *Ralstonia pickettii, Ralstonia pseudosolanacearum*, *Ralstonia solanacearum*, and *Ralstonia syzygii* (List of Prokaryotic Names with Standing in Nomenclature, 2021). Although *Ralstonia* species are usually environmental bacteria, *R. pseudosolanacearum*, *R. solanacearum*, and *R. syzygii* have been described as plant pathogens (Safni et al. 2014). *R. insidiosa*, *R. mannitolilytica*, *R. pickettii* have occasionally been reported as pathogens in a variety of human infections (Ryan and Adley 2014), but their pathogenicity is considered to be low. *R. mannitolilytica* is the species most often found in cystic fibrosis (CF) patients, and these patients may become chronically infected with the bacterium (Green et al. 2017; Green and Jones 2018). Its prevalence in CF patients is less than 5%, but has been increasing in the last decade. This increase may be largely due to improved diagnostics, but other factors, e.g., improved awareness or a true increase, may also play a role (Burns et al. 1998; Coenye et al. 2002; Daxboeck et al. 2005; Lipuma 2010; Green et al. 2017). *Ralstonia* species are generally multi-resistant to antibiotics; *R. mannitolilytica* appears to be more resistant than *R. pickettii* and *R. insidiosa* (Green et al. 2017). Little is known about the transmission of *Ralstonia* spp., but outbreaks mostly seem to be caused by contaminated solutions, however, person-to-person transmission cannot be concluded. (Ryan and Adley 2013; Coman et al. 2017; Green et al. 2017; Green and Jones 2018).

Our knowledge of *Ralstonia* spp., their role in human infection is still limited and the taxonomic relations between the different species. Therefore, in this study, we determined and analysed the genomes of 18 *Ralstonia* strains recovered from patient specimens.

## Materials and methods

### Isolates

A total of 18 strains were included in the study. These strains were recovered between 2003–2016 from 17 patients in three different countries: Spain (n = 6), the Netherlands (n = 10), and the USA (n = 2) (Table 1). Matrix-assisted laser desorption/ionization time of flight (MALDI-TOF, Bruker) had previously identified seven strains as *R. pickettii* and four as *R. mannitolilytica* (scores ≥2.0)*.* Seven strains were only identified as *Ralstonia* species the genus level (top ten scores 1.700–2.000); of these, five had the highest (MALDI-TOF) similarity with *R. mannitolilytica*, one with *R. pickettii,* and one with *R. insidiosa*.

**Table 1:** Source, MICs (mg/L), and resistance genes

Strain	Species WGS	Country	Isolate source	Aztreonam	Ceftazidime	Ciptrofloxacin	Colistin
16-551634	*R. pickettii*	The Netherlands	Blood culture	128	16	0.12	> 16
16-551636	*R. pickettii*	The Netherlands	Blood culture	256	8	0.12	> 16
16-543514	*R. pickettii*	The Netherlands	Blood culture	256	16	0.06	> 16
16-551632	*R. pickettii*	The Netherlands	Blood culture	256	8	0.12	> 16
16-551637	*R. pickettii*	The Netherlands	Blood culture	> 256	8	0.06	> 16
16-543504	*R. pickettii*	The Netherlands	Blood culture	> 256	32	0.12	> 16
16-551631	*R. pickettii*	The Netherlands	Furunkulosis	> 256	32	0.25	> 16
16-551635	*R. pickettii*	The Netherlands	Spondylodicitis	> 256	32	0.25	> 16
16-535633	*R. mannitolilytica*	Spain	CF	> 256	64	> 32	> 16
16-535634	*R. mannitolilytica*	Spain	CF	> 256	256	4	> 16
16-535635	*R. mannitolilytica*	Spain	CF	> 256	64	0.25	> 16
16-545260	*R. mannitolilytica*	USA	CF	> 256	32	> 32	> 16
16-545261	*R. mannitolilytica*	USA	CF	> 256	16	32	> 16
16-535632	*R. mannitolilytica*	Spain	CF	256	16	32	> 16
16-535638	*R. mannitolilytica*	Spain	CF	> 256	16	0.12	> 16
16-543498	*R. mannitolilytica*	The Netherlands	Blood culture	> 256	32	0.12	> 16
16-551633	*R. insidiosa*	The Netherlands	Blood culture	> 256	16	0.12	> 16
16-535637	*R.* new spp.	Spain	CF	> 256	16	0.12	> 16

Strain	Co-trimoxazole	Imipenem	Meropenem	Tobramycin	Resistance genes	Group^1^	
16-551634	<=0.06	<=0.12	0.5	64	*bla*_OXA-22_ family, *bla*_OXA-60_ family	E2	
16-551636	<=0.06	16	32	64	*bla*_OXA-22_ family, *bla*_OXA-60_ family	E2	
16-543514	<=0.06	8	64	64	*bla*_OXA-22_ family, *bla*_OXA-60_ family	E2	
16-551632	<=0.06	8	16	16	*bla*_OXA-22_ family, *bla*_OXA-60_ family	E2	
16-551637	<=0.06	8	32	16	*bla*_OXA-22_ family, *bla*_OXA-60_ family	E2	
16-543504	<=0.06	8	> 64	2	*bla*_OXA-22_ family, *bla*_OXA-60_ family	E1	
16-551631	<=0.06	2	8	16	*bla*_OXA-22_ family, *bla*_OXA-60_ family	E1	
16-551635	<=0.06	8	8	16	*bla*_OXA-22_ family, *bla*_OXA-60_ family	E1	
16-535633	8	64	> 64	> 128	*bla*_OXA-22_ family, *bla*_OXA-60_ family	D2	
16-535634	8	128	> 64	128	*bla*_OXA-22_ family, *bla*_OXA-60_ family	D2	
16-535635	0.12	32	64	128	*bla*_OXA-22_ family, *bla*_OXA-60_ family	D2	
16-545260	32	16	> 64	> 128	*bla*_OXA-22_ family, *bla*_OXA-60_ family, *aadA2*, *ant(2'')-Ia*, *aph(6)-Id*, *cmlA1*, *strA*, *sul1*	D2	
16-545261	2	4	64	128	*bla*_OXA-22_ family, *bla*_OXA-60_ family, *aadA2*, *ant(2'')-Ia*, *aph(6)-Id*, *cmlA1*, *strA*, *sul1*	D2	
16-535632	1	32	> 64	128	*bla*_OXA-22_ family, *bla*_OXA-60_ family	D2	
16-535638	0.12	32	> 64	32	*bla*_OXA-22_ family, *bla*_OXA-60_ family	D1	
16-543498	0.12	64	> 64	64	*bla*_OXA-22_ family, *bla*_OXA-60_ family	D1	
16-551633	<=0.06	16	32	64	*bla*_OXA-22_ family, *bla*_OXA-60_ family	G	
16-535637	0.25	8	8	32	*bla*_OXA-22_ family, *bla*_OXA-60_ family	F	

^1^Groups defined on basis of the ANIb (Fig. 2 and supplementary Fig. 1)

### Whole genome sequencing

Bacterial DNA was purified using the Qiacube with the DNeasy Blood & Tissue kit with the enzymatic lysis protocol (Qiagen, Carlsbad, CA) and used to prepare a library for sequencing with the MiSeq or NextSeq platforms (Illumina, San Diego, CA), using the Nextera XT library prep kit (Illumina). Subsequently, sequencing was performed on an Illumina NextSeq platform, using the 2 x 150 bp sequencing kit. All reads were trimmed with seqtk trimfq version 1.3 (Seqtk 2019), with an error rate threshold of 0.001. Nextera transposase sequences were removed with trim-galore version 0.5.0 (Trim-galore 2019). Contigs were assembled with SPAdes genome assembler v.3.11.1, and contigs larger than 500 bp with at least 10x coverage were analysed further.

### Genome analysis

In total, 18 genomes were used to create a core genome multi-locus sequence typing (cgMLST) scheme for the *Ralstonia* strains. The genomes of 7 strains available to the public were used as reference to build a reference cgMLST scheme for this genus with Ridom SeqSphere software version 5.0.0 (Mellmann et al. 2016) (Supplementary table 1). The genomes were aligned with BLAST to find homologous genes; a query gene in the reference cgMLST scheme was considered to have a homologue if the two genes completely overlapped with an identity of at least 90% (BLAST version 2.2.12. (Altschul et al. 1990)).

The evolutionary history for the OXA ß-lactamases was inferred by using the Maximum Likelihood method for amino acid sequences, which was based on the JTT matrix-based model in MEGA-X version 10.0.4 (Jones et al. 1992). The tree with the highest log likelihood is shown. The percentage of 500 bootstrap trees in which the associated taxa clustered together is shown next to the branches. Initial tree(s) for the heuristic search were obtained automatically by applying Neighbor-Join and BioNJ algorithms to a matrix of pairwise distances estimated, using a JTT model, and then selecting the topology with superior log likelihood value. The tree is drawn to scale, with branch lengths measured in the number of substitutions per site. Evolutionary analyses were conducted in MEGA-X (Kumar et al. 2018). The assembled contigs were annotated using RAST version 2.0 (Overbeek et al. 2014).

The evolutionary history of the 16S rDNA sequences was inferred by using the Maximum Likelihood method based on the Tamura-Nei model in MEGA-X version 10.0.4 (Tamura and Nei 1993). The percentage of 500 bootstrap trees in which the associated taxa clustered together is shown next to the branches. Initial tree(s) for the heuristic search were obtained automatically by applying Neighbor-Join and BioNJ algorithms to a matrix of pairwise distances estimated using the Maximum Composite Likelihood (MCL) approach, and then selecting the topology with superior log likelihood value. Evolutionary analyses were conducted in MEGA-X (Kumar et al. 2018).

To obtain a better insight into the relationship between the 18 strains and other *Ralstonia* species, an ANIb (Average Nucleotide Identity based on BLAST) analysis was performed. In this analysis, the 57 genome sequences available from GenBank were included (Supplementary table 2), as well as those of the type strains for *R. insidiosa*, *R. pickettii*, *R. solanacearum* and the only available draft genome sequence for *R. syzygii* species. For the other species, no whole genome sequences (WGS) of the type strains were available. Average nucleotide identity (ANI) among the genomes was calculated using ANIb algorithm of the pyani tool version 0.2.3 (Pritchard et al. 2016), which uses nucleotide BLAST alignment (version 2.2.28+) for whole genome alignment. Genomes were split into genomic fragments of 1020 bases long; pairwise alignments of the fragments of each genome were performed and ANI was calculated as the percentage of nucleotide identity for matching regions of all genomes. In biclustering analysis of ANI scores, complete linkage was used as a hierarchical clustering method with the Euclidean distance metric. A heatmap of all genomes was generated using biclustering, with a colour scale bar showing the pairwise ANI score. A cut-off of 0.95 was used to define groups, which is also the cut-off used to define species (Richter and Rosselló-Móra 2009).

### Antimicrobial susceptibility testing

Minimum inhibitory concentrations (MICs) were determined by the standard ISO broth microdilution method with frozen panels (Trek Diagnostic Systems, Westlake, OH) using EUCAST criteria (European Committee on Antimicrobial Susceptibility Testing, 2019). The antimicrobial agents and the concentration range tested were as follows: aztreonam (0.25–256 mg/l); ceftazidime (0.25–256 mg/l); ciprofloxacin (0.03–32 mg/l); colistin (0.25–16 mg/l); tobramycin (0.125–128 mg/l); imipenem (0.125–128 mg/l); meropenem (0.06–64 mg/l); and trimethoprim/sulfamethoxazole (co-trimoxazole) (0.06–32 mg/l).

## Results

### Sequence characteristics

Identification by MALDI-TOF at the central laboratory showed 8 *R. mannitolilytica*, 9 *R. pickettii*, and a strain with a genus level identification with *R. insidiosa* as best match. This coincided with speciation based on WGS, with one exception: isolate 535637, identified as *R. pickettii* by MALDI-TOF, could not be linked to any known species by WGS.

Sequencing and assembly of whole genomes for *R. mannitolilytica*, *R. pickettii*, *R. insidiosa*, and the new *Ralstonia* spp. resulted in at least 45-fold coverage for the 18 strains with a maximum of 117 contigs and a genome size of approximately 4.8–6.4 MB, indicating good quality sequences (Supplementary table 3). The average genome size differed considerably: 5,272,894, 4,932,406, 6,385,888, and 5,676,110 bp using contigs ≥ 1000 bp with an average GC content of 65.85, 63.68, 63,25, 63.32%, for *R. mannitolilytica*, *R. pickettii*, *R. insidiosa*, and the new *Ralstonia* spp., respectively.

### Position in the genus *Ralstonia*

**Fig. 1:**
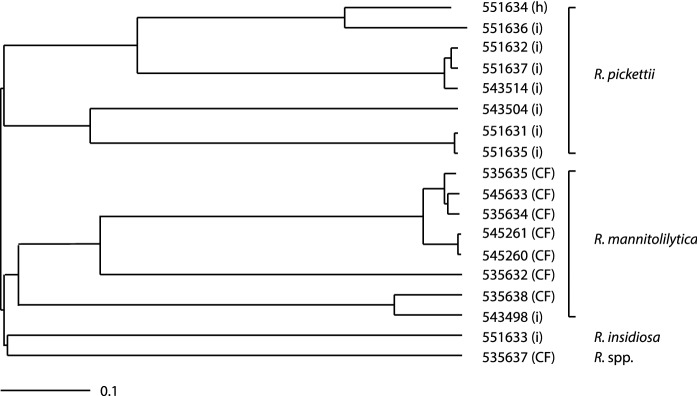
Phylogenetic tree of 18 *Ralstonia* strains. The tree was created using presence/absence profile of the homologues of 517 core genes (cgMLST) (see Methods section for more details) in 18 query genomes. The letter between brackets indicates the source of the strain. CF: cystic fibrosis; h: human strain but not information on infection or carriership; i: infection

A cgMLST based on 517 genes showed long branches between strains belonging to either *R. pickettii* or *R. mannitolilytica* (Supplementary table 4). These branches were nearly as long as the branches of *R. mannitolilytica* and *R. insidiosa*. The long branch for isolate 535637 and whole genome alignments suggested that this isolate belongs to a new species (Fig. 1).

**Fig. 2:**
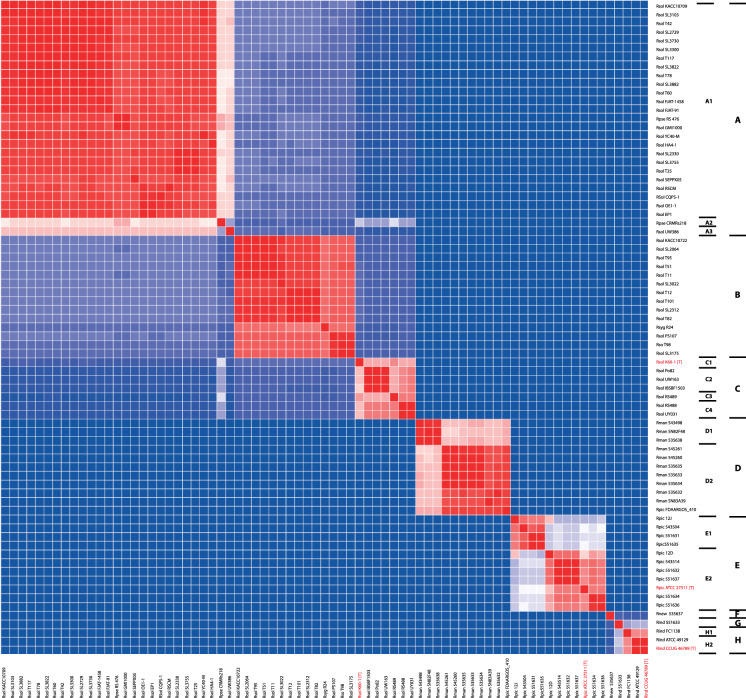
Heatmap based on percentage of ANIb for all 18 *Ralstonia* strains that were analysed in this study. The color shows the percentage of ANIb between any two strains starting from blue (80%) through white to red (100%)(see Methods section for more details). The species names are derived from the GenBank data or MALDI-TOF. At the left the original identification of the strains; Rind: *R. insidiosa*; Rman: *R. mannitolilytica;* Rpic: *R. pickettii*; Rpse: *R. pseudosolanacearum*; Rsol: *R. solanacearum*; Rsyz: *R. syzygii*. Type strains are indicated in red and by [T] behind the strain identification. The (sub)groups (see text) are indicated at the right. The (sub)groups (see text) are indicated at the right. For details about strains and the similarity between strains see Supplementary Fig. 1

An ANIb analysis was performed which only included the type strains for *R. insidiosa*, *R. pickettii*, *R. solanacearum* (Fig. 2, Supplementary Fig. 1). For the other species no WGS of the type strains was available (Supplementary table 2). A cut-off of 0.95 was used to define groups of strains that clustered together, which is also the cut-off to define species (Richter and Rosselló-Móra 2009). The groups were provisionally given letter indications (A-H) with eight groups; possible subgroups (cut-off ≤0.96 within a group) were indicated with numeric suffixes. Group A contained three subgroups, group C consisted of four subgroups, and groups D, E, and H were composed of two subgroups each. Groups A-C contained strains previously identified as *R. solanacearum* or *R. pseudosolanacearum* (two strains: one in A1 and one in A2), and also the only available WGS of a *R. syzygii* strain (in group B). The type strain for *R. solanacearum* clustered with group C and was the sole representative of subgroup C1 with a maximum ANIb score of 0.97 compared to the other strains in group C. Group D contained strains previously identified as *R. mannitolilytica*.

Strains identified as *R. pickettii* clustered in group E. This group seemed to be comprised of two subspecies (or possibly true species) with the type strain clustering in subgroup E2. However, *R. pickettii* FDAARGOS-410 clustered with *R. mannitolilytica* in group D2. Strains previously identified as *R. insidiosa* were present in group H. Strain 551633, which was identified as only to the genus level with MALDI-TOF and the best match with *R. insidiosa*, was the only representative of group G. The type strain for *R. insidiosa* clustered in group H2.

**Fig. 3:**
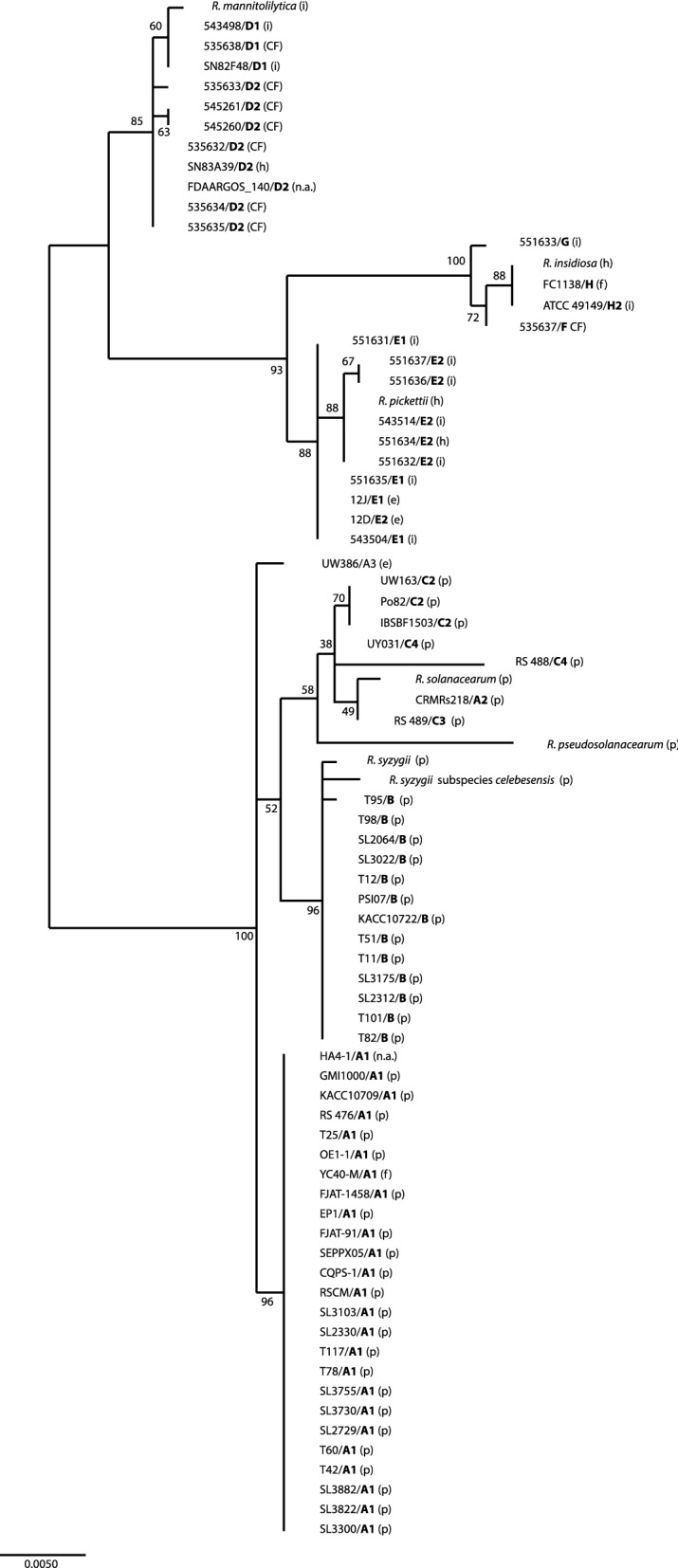
Maximum likelihood tree for the 16S rDNA sequences of the strains used in the ANIb. The analysis involved 78 nucleotide sequences. There were a total of 1395 positions in the final dataset. The tree with the highest log likelihood (-2740.49) is shown. Strain identifications/(sub)groups are indicated in bold; type strains are indicated by the species names; numbers at branch points show bootstrap values. The letter between brackets indicates the source of the strain. CF: cystic fibrosis; e: environment; h: human strain but not information on infection or carriership; i: infection; n.a.: no data available; p: plant pathogen

In addition, 16S rRNA gene sequences were compared (Fig. 3). The phylogenetic tree showed that the strains of subgroup A1 were all identical, as were nearly all strains in group B. The latter group included the type strains for *R. syzygii* and *R. syzygii* subspecies *celebesensis*. The group D strains fell into two subclusters that matched subgroups D1 and D2. The type strain for *R. mannitolilytica* matched best with subgroup D1 (1 SNP and 1–2 SNPs with D2 (Supplementary Table 5)). It should be noted that *R. pickettii* FDAARGOS-410 also clustered in subgroup D2. A similar division into two subgroups was seen in group E, with the exception of strain 12D; strain 12D clustered among the E1 subgroup by 16S rDNA analysis, whereas it clustered in subgroup E2 in the ANIb (Fig. 2, Supplementary figure 1). The type strain for *R. pickettii* clustered in subgroup E2 (1 SNP and 2 SNPs with E1). The sequences for the groups E-G all clustered together with the type strain for *R. insidiosa* (0 SNPs difference with H, 2 with F and 3 with G). The strains of group C clustered more loosely, and clustered with the single representative of the A2 subgroup. The 16S rRNA gene sequence of the A2 strain was identical to that of the C3 strain and the type strain for *R. solanacearum* clustered most closely to these two strains (2 SNPs). The closest relation of the *R. pseudosolanacearum* type strain sequence was also group C, but with a distance of at least 18 SNPs (Supplementary Table 5).

Our 18 isolates belonged to the (sub)groups D1, D2, E1, E2, F or G (Table 1, Fig. 2, Supplementary Fig. 1).

**Fig. 4:**
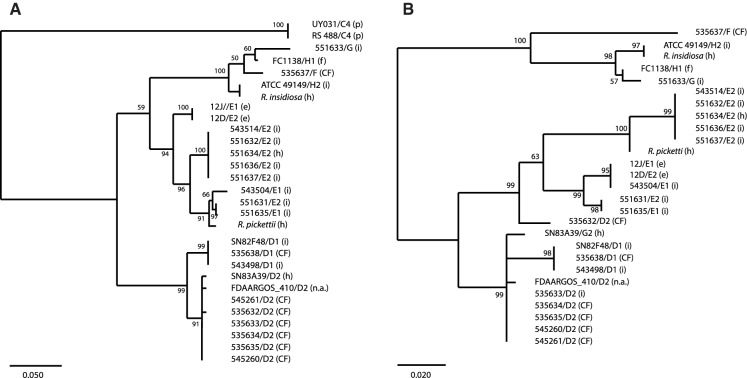
Maximum likelihood tree for the OXA ß-lactamase sequences of the strains used in the ANIb. Panel A: OXA-22 family; panel B: OXA-60 family. The analysis involved 29 amino acid sequences. There were a total of 279 positions in the final dataset. The OXA-22 family analysis involved 29 amino acid sequences. There were a total of 279 positions in the final dataset. The OXA-60 family analysis involved 27 amino acid sequences. There were a total of 271 positions in the final dataset. Strain identifications/(sub)groups are indicated; type strains are indicated by the species names (when a sequence was available); numbers at branch points show bootstrap values. The letter between brackets indicates the source of the strain. CF: cystic fibrosis; e: environment; h: human strain but not information on infection or carriership; i: infection; n.a. no data available; p: plant pathogen

Finally, the OXA-22 and OXA-60 family ß-lactamase sequences were analysed. The genes for these ß-lactamases were not present in (sub)groups A, B, C2, and C3, nor in the type strains for *R. solanacearum*, *R. pseudosolanacearum*, *R. syzygii*, and *R. syzygii* subspecies *celebesensis*. In addition, the gene for the OXA-60 family ß-lactamase was also absent in group C4. The clustering of the OXA-22 and OXA-60 ß-lactamase amino acid sequences of the other groups largely followed the WGS-based grouping (Fig. 4a and b. However, some of the E1 and E2 strains clustered together. In addition, the sequence of strain 535632 (from subgroup D2) was more closely related to the group E strain sequences.

### Antibiotic resistance and patient characteristics

All strains showed high MICs against colistin (>16 mg/l) and aztreonam (≥256 mg/l) with one exception (Table 1). Ciprofloxacin MICs were generally low (≤0.12 mg/l), although four *R. mannitolilytica* strains had MICs of ≥32 mg/l. Co-trimoxazole also generally showed low MICs (≤1 mg/l), but with three *R. mannitolilytica* strains having MICs ≥8 mg/l; these three strains also exhibited high MICs against ciprofloxacin (Table 1). MICs against the other antibiotics varied, but were generally high, in particular for tobramycin. All strains harboured the OXA-22 and OXA-60 ß-lactamase family genes, ResFinder only detected acquired resistance genes in two very similar (but not identical) *R. pickettii* strains from the same patient, namely: *aadA2*, *strA*, *ant(2'')-Ia*, *aph(6)-Id*, *cmlA1* and *sul1*. The first four genes encode resistance against aminoglycosides, the last two against chloramphenicol and sulfonamide, respectively. The genes *aadA2*, *ant(2'')-Ia*, *cmlA1* and *sul1* are part of a class I integron.

*Ralstonia mannitolilytica* was the *Ralstonia* species most often associated with CF: seven of the eight strains recovered from people with CF (all from respiratory samples) were *R. mannitolilytica* and one was the novel *Ralstonia* species. *R. pickettii* was the species most often found in other patients: of the ten non-CF strains, eight were *R. pickettii*, one was *R. mannitolilytica* and one was *R. insidiosa*. Six of the ten strains were recovered from blood cultures, all in patients who had intravascular catheters; likely this represented cases of IV-catheter related bacteraemia. Two cases were deemed contaminants from arthroplasties infected with different micro-organisms and were not treated with antibiotics against *Ralstonia* spp. Furthermore, there was one case of spondylodiscitis (co-infection with *Escherichia coli*) and one case of a furunculosis abscess (co-infection with *Staphylococcus aureus*); in both these cases *R. pickettii* was treated with antibiotics (Table 1, Supplementary table 3).

### Comparison of gene functions

RAST annotation of our strains showed that the number of coding sequences for *R. pickettii* was higher than for *R. mannitolilytica* (range 4613–5137 and 5009–5498, respectively) (Supplementary Table 3). The numbers of RNA encoding genes, as well as most other classes of genes, in general do not differ much between the different species within the genus. An exception here were the genes in the category “virulence, disease and defense”, where an almost two-fold difference in the number of genes was observed between the *R. insidiosa* strain 551633 and *R. mannitolilytica* 535635. The difference was nearly completely attributable to the subcategory “resistance to antibiotic and toxic compounds” which contained 171 genes in the *R. insidiosa* strain. The subgroup D1 *R. mannitolilytica* strains also had a higher number of genes in this category compared to the D2 strains. In this category, genes conferring resistance to heavy metals, bleomycin, polymyxin, fosfomycin, albicidin, and organic peroxides, and efflux pumps were present. Differences between strains were mostly found in the resistance against bleomycin, polymyxin, fosfomycin and albicidin. All strains harboured a gene encoding a bacteriocin defense protein.

*Ralstonia mannitolilytica* 535632 and *R. pickettii* 551635 had more than 10 genes assigned as putative phage genes; the other strains had eight or less assigned. The majority of these genes were present in contiguous sequences. Transposon-related sequences were identified in one *R. mannitolilytica* (535638) and all strains of the other species. Plasmid-related sequences were not identified in any of the species.

The subgroups in *R. mannitolilytica* and *R. pickettii* showed limited differences in the number of genes for the different categories defined by RAST: subgroup D1 within *R. mannitolilytica* had a lower number of genes in the category “amino acid and derivatives” compared to D2 (range 359–372 and 402–416, respectively) and *R. pickettii* subgroup H1 had less genes in the category “membrane transport” (range 125–132 and 149–193, respectively).

## Discussion

In this study, WGS was performed on 18 clinical *Ralstonia* strains; the results were compared to available *Ralstonia* WGS data and available genomic data of the *Ralstonia* type strains. It was found that the diversity within the *Ralstonia* genus is higher than expected based on ANIb. The taxonomy will need reorganization and we suggest the addition of a number of novel species and possibly subspecies based on a cut-off 95% for species and results from the ANIb (Fig. 2, Supplementary Fig. 1) (Richter and Rosselló-Móra 2009).

*Ralstonia mannitolilytica* was the species most often found in respiratory samples from CF patients, with *R. pickettii* being the most common species in other infections. The majority of case reports of *Ralstonia* infections in literature are with *R. pickettii*, but *R. mannitolilytica* is also frequently reported as species (Prior et al. 2017).

All our clinical strains harboured OXA-22 and/or OXA-60 family ß-lactamases, and the ß-lactam antibiotics tested all had high MICs. Acquired antibiotic resistance genes were only found in two CF strains from a single patient; these two strains were indeed amongst the most resistant. The MICs of colistin and tobramycin were high for all strains. Ciprofloxacin and co-trimoxazole had low MICs for *R. pickettii*, *R. insidiosa* and the putative novel *Ralstonia* species. However, these two antibiotics mostly had high MICs for *R. mannitolilytica.* This might be due to adaptations of the strains in the lung of CF patients, who are often treated with these antibiotics for chronic lung infection. Of note, the single *R. mannitolilytica* strain isolated from a patient who did not suffer from CF had low MICs for both ciprofloxacin and co-trimoxazole, possibly indicating that this resistance is acquired during chronic infection.

A cgMLST-based phylogenetic tree built with the 18 strains (Fig. 1) showed long branches, suggesting the presence of subspecies or novel species. Subsequently, an ANIb analysis was performed to further elucidate the position of our strains and compare them to sequenced strains in the GenBank database. Unfortunately, only the type strains of *R. insidiosa*, *R. pickettii* and *R. solanacearum* could be included in the ANIb, as WGS was not available for the other type strains. Based on a cut-off of 0.95, the ANIb data found eight different groups or species (A-H) (Richter and Rosselló-Móra 2009). Furthermore, a number of subgroups were identified that could possibly be considered as separate species or subspecies. (Fig. 2, Supplementary Fig. 1). For some (sub)groups the WGS of only one strain was available, which hampers the ability to delimit defined species and identify novel species. Nevertheless, by combining the ANIb data with 16S rDNA phylogenetic tree data (Fig. 3) a number of proposals to adapt the taxonomy of the genus *Ralstonia* can be made. Subgroup A1 appears a separate species that has not been described before, despite the fact that almost all strains have been identified previously as *R. solanacearum*, as the *R. solanacearum* type strain 16S rDNA sequence matches more closely with other groups (Supplementary table 5). Subgroup A1 also contains a *R. pseudosolanacearum*, but 16S rRNA gene sequence of the type strain of this species shows 20 SNPs when compared to the 16S rRNA gene sequences of other group members. Subgroup A2, although being on the border of a different species based on the ANIb and containing only a single strain (previously identified as *R. pseudosolanacearum*), may be a novel species, as it has a different 16S rDNA sequence (18 SNPs difference) compared to the *R. pseudosolanacearum* type strain sequence. Subgroup A3 contains only a single strain, previously identified as *R. solanacearum*, and has an ANIb score of 0.96 compared to the A1 strains, but its 16S rDNA sequence differs from the other strains in group A to the extent that it also may be a novel (sub)species (Supplementary table 5).

Group B strains are *R. syzygii* based on the ANIb and clustering of the 16S rDNA sequences with 1–2 SNPs. However, this is based on the assumption that the R*. syzygii* strain used in the ANIb previously was identified correctly.

The data for group C, with all strains previously identified as *R. solanacearum*, are less clear, but the ANIb and 16S rDNA sequence data support this identification. Within this species four subspecies could be suggested. Additionally, subgroup C4 encodes an OXA-22 family ß-lactamase, which supports a separate status from the other group C strains.

Group D consists of only *R. mannitolilytica* strains, which is in agreement with the prior identification of the strains. The ANIb, 16S rDNA, OXA-22 and OXA-60 ß-lactamase sequence clustering (Fig. 4) support that these may be two subspecies.

Based on ANIb data, the group E strains should be considered as at least two subspecies, and possibly as two species. The type strain for *R. pickettii* clusters with subgroup E2. However, some mixing of E2 and E1 subgroup strains is observed for both 16S rDNA and ß-lactamase strains. This may indicate that genetic exchange has taken place between these subgroups. Genetic exchange may also have taken place between group E and group D strains: strain 535632 (group D) clusters with group E strains for the OXA-60 family ß-lactamase (Fig. 4b). However, (*in vitro*) evidence for exchange of genetic material between *Ralstonia* species is not available.

The strains in group F and G can be considered novel species based on ANIb clustering, but both are closely related to *R. insidiosa* based on 16S rDNA analysis. The type strain for *R. insidiosa* clusters with subgroup H2; in our proposal group H strains belong to *R. insidiosa*. OXA-22 and OXA-60 ß-lactamase sequence clustering support the suggestion of three species. A finding of an isolate that belonged to an unnamed species was recently also described by Coward et al. (Coward et al. 2020).

Two closely related *R. pickettii* strains contained identical acquired antibiotic resistance genes (Table 1, Fig. 1): *aadA2*, *ant(2'')-Ia*, *cmlA1* and *sul1*, which are part of a class I integron. Analysis of the WGS indicates the presence of a second class I integrase, but the short-read length limited the assembly of repeat sequences. The gene cassettes in this putative second integron could therefore not be identified.

All strains contained OXA ß-lactamases; most probably these genes are important in the environmental niches where *Ralstonia species* are normally found. However, ß-lactamase formation may play an important role in polymicrobial infections or the cystic fibrosis lung where ß-lactamase-producing species or strains protect ß-lactam antibiotic-susceptible species or strains. A study by Sherrard et al demonstrated this effect *in vitro* using a ß-lactamase-producing *Prevotella* strain and a susceptible *Pseudomonas aeruginosa* from CF patients (Sherrard et al. 2016). A co-culture of the two strains in the presence of 32 mg ceftazidime l^-1^, a clinical relevant concentration, showed a viable *P. aeruginosa* count of 1.63 x 10^6^ colony forming units (CFU)/ml compared to <x10^2^ CFU/ml for a monoculture. Resistance development in the *P. aeruginosa* strain was excluded. Although the *in vivo* situation is more complex, with multiple species present, this effect may also be relevant in infection. This was demonstrated in a pneumonia model in mice using ß-lactamase producing *Moraxella catarrhalis* and penicillin-susceptible pneumococci. None of the mice survived when pneumococci were inoculated, but all mice survived with a curative dose of penicillin. Co-inoculation *M. catarrharalis* and penicillin-susceptible pneumococci resulted in the death of a significant number of mice. This effect seemed to be dependent on the dose of ß-lactamase producing *M. catarrharalis* (Hol et al. 1994).

Genes classified as “virulence, disease and defense” and in particular the subcategory “resistance to antibiotic and toxic compounds” probably contribute to survival in natural habitats, but a role in survival in the human lung cannot be excluded. The *Ralstonia* strains generally lacked virulence factors recognized in other bacterial species, indicating that they are likely opportunistic pathogens.

Annotation showed only limited evidence for mobile elements in the genomes of the 18 clinical strains. Phage protein genes were found in sixteen of the eighteen strains, but the majority had less than 8 of such genes, whereas lysogenic phages generally encode at least 40 proteins. This suggests that lysogenic phages may be present, but that they would differ considerably from known phages. Evidence for a transposon was only found in *R. pickettii* strains and one *R. mannitolilytica*, indicating that these are not very common or different from known transposons. No evidence for plasmids was found, also suggesting either their absence or a significant difference from described plasmids.

In summary, the taxonomy of the genus *Ralstonia* needs revision, as several new species and subspecies might be identified based on whole genome sequence data. We suggest that group A is a novel species which may contain two additional (sub)species. Group B is probably *R. syzygii* and group C *R. solanacearum*. Group D is composed of *R. mannitolilytica* and may include two subspecies. Group E strains belong to *R. pickettii*, but this group may comprise two subspecies. The strains in groups F and G might be considered novel species, although both are closely related to *R. insidiosa*. The type strain for *R. insidiosa* clusters with subgroup H2; in our suggestion group H strains therefore belong to *R. insidiosa*.

A requirement that the formal proposal of a new (culturable) species includes the whole genome sequence of the corresponding type strain would help to correctly identify strains of the species and to distinguish novel (sub)species.

MIC data for *Ralstonia* species are limited and reports with larger number of isolates are only available for *R. pickettii* and *R. insidiosa*. One study by Ryan and Adley involved 53 *R. pickettii* isolates from diverse sources including environmental isolates form different culture collections (Ryan and Adley, 2013). The other study investigated 39 *R. pickettii* clinical isolates that were collected worldwide (Sader and Jones 2005). For aztreonam our isolates showed comparable MICs to both studies and for ceftazidime we found somewhat higher MICs. For meropenem and ciprofloxacin we found somewhat higher MICs based on MIC_50_ and MIC_90_ data. We observed somewhat lower MICs for ciprofloxacin and co-trimoxazole. For meropenem comparable values were compared to the first study and somewhat lower compared to the worldwide study. Tobramycin and imipenem results were only reported for the worldwide study and our results were comparable and slightly higher, respectively. The other antibiotics we tested were not reported in the earlier studies. It should be noted however, that we had only a few isolates and also sources and regions were not comparable. The high levels of resistance against ß-lactam antibiotics is usually attributed to the presence of inducible OXA-type ß-lactamases. However, we had one isolate (16-551634) with a low MIC for carbapenems (Table 1) indicating either a non-functional ß-lactamase or induction mechanism (Nordmann et al. 2000; Girlich et al. 2004).

## Supplementary Information

Below is the link to the electronic supplementary material.

Supplementary file1 (XLSX 11 kb)

Supplementary file2 (XLSX 14 kb)

Supplementary file3 (XLSX 14 kb)

Supplementary file4 (XLS 114 kb)

Supplementary file5 (XLSX 10 kb)

Supplementary file6 (PDF 16470 kb)

## Data Availability

This whole-genome shotgun project has been deposited at ENA under BioProject ID: PRJNA611754; the BioSample IDs for the strains are: strain 551632: SAMN14345102; strain 551635: SAMN14345103; strain 551634: SAMN14345104; strain 551637: SAMN14345105; strain 551636: SAMN14345106; strain 551631: SAMN14345107; strain 535637: SAMN14345108; strain 543504: SAMN14345109; strain 543514: SAMN14345110; strain 535632: SAMN14345111; strain 535634: SAMN14345112; strain 535633: SAMN14345113; strain 551633: SAMN14345114; strain 535635: SAMN14345115; strain 545260: SAMN14345116; strain 545261: SAMN14345117; strain 535638: SAMN14345118; strain 543498: SAMN14345119.
