# Supplementary material for: Characterization of clinical Ralstonia strains and their taxonomic position
Source: Antonie Van Leeuwenhoek. 2021 Aug 31;114(10):1721–33. doi: 10.1007/s10482-021-01637-0 (PMC8448721; doi:10.1007/s10482-021-01637-0)
Supplement: Supplementary file 6 — Supplementary file6 (PDF 16470 kb) [file 10482_2021_1637_MOESM6_ESM.pdf]

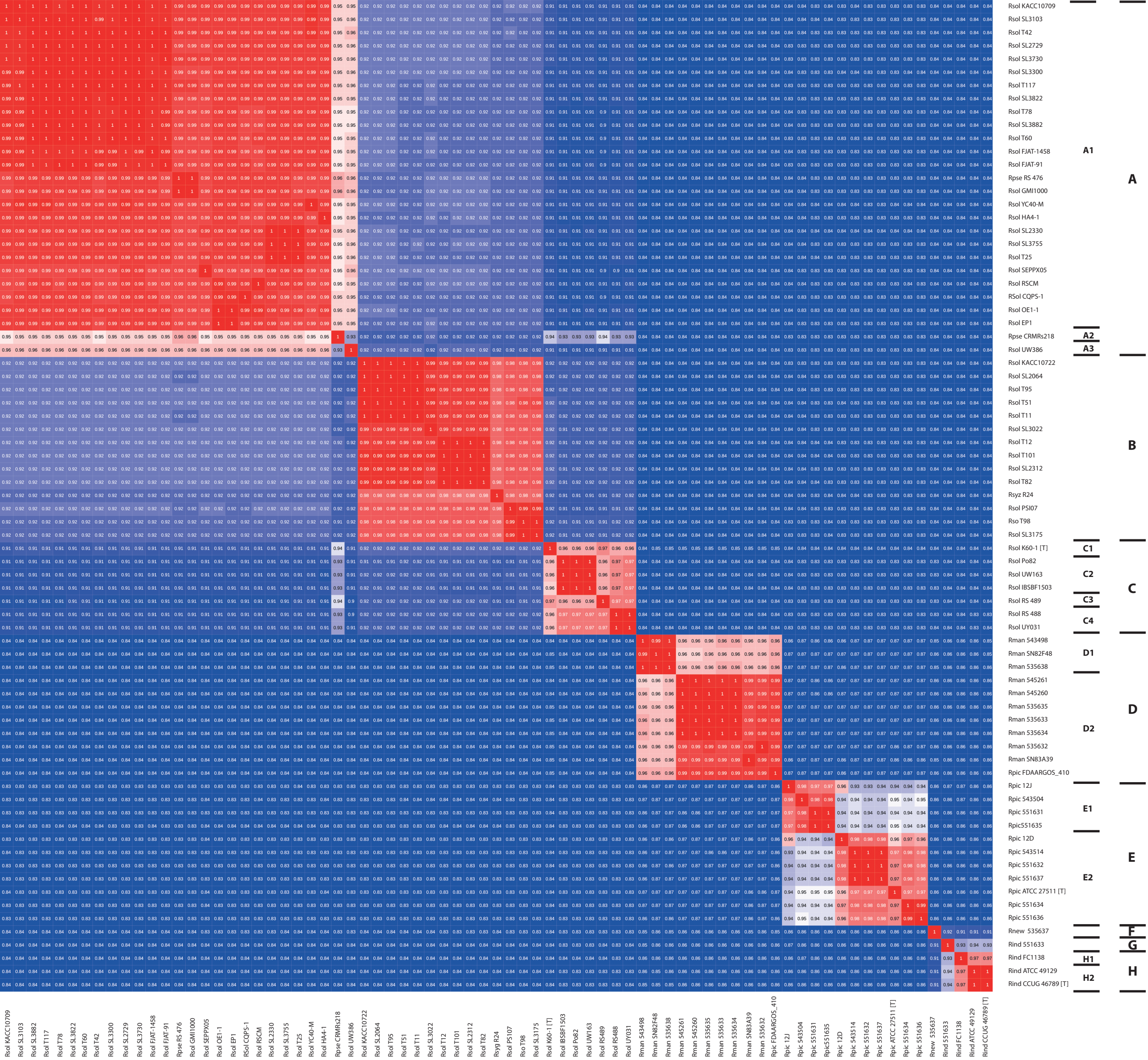


**Supplementary figure 1. Heatmap based on percentage of ANIb for all 18 *Ralstonia* strains that were analyzed in this study.** The color shows the percentage of ANIb between any two strains starting from blue (80%) through white to red (100%). The species names are derived from the GenBank data or MALDI-TOF. Rind: *R. insidiosa*; Rman: *R. mannitolilytica;* Rpic: *R. pickettii*; Rpse: *R. pseudosolanacearum*; Rsol: *R. solanacearum*; Rsyz: *R. syzygii*. Type strains are indicated by [T] behind the strain identification. The (sub)groups (see text) are indicated at the right.
